# Cholesterol metabolism in tumor microenvironment: cancer hallmarks and therapeutic opportunities

**DOI:** 10.7150/ijbs.92274

**Published:** 2024-03-17

**Authors:** Wen Jiang, Wei-Lin Jin, A-Man Xu

**Affiliations:** 1 Department of General Surgery, The First Affiliated Hospital of Anhui Medical University, Hefei 230022, P. R. China.; 2 Institute of Cancer Neuroscience, Medical Frontier Innovation Research Center, The First Hospital of Lanzhou University, Lanzhou 730000, P. R. China.; 3 Anhui Public Health Clinical Center, Hefei 230022, P. R. China.

**Keywords:** antitumor immunity, cholesterol homeostasis, drug repurposing, metastasis, statin, tumor microenvironment

## Abstract

Cholesterol is crucial for cell survival and growth, and dysregulation of cholesterol homeostasis has been linked to the development of cancer. The tumor microenvironment (TME) facilitates tumor cell survival and growth, and crosstalk between cholesterol metabolism and the TME contributes to tumorigenesis and tumor progression. Targeting cholesterol metabolism has demonstrated significant antitumor effects in preclinical and clinical studies. In this review, we discuss the regulatory mechanisms of cholesterol homeostasis and the impact of its dysregulation on the hallmarks of cancer. We also describe how cholesterol metabolism reprograms the TME across seven specialized microenvironments. Furthermore, we discuss the potential of targeting cholesterol metabolism as a therapeutic strategy for tumors. This approach not only exerts antitumor effects in monotherapy and combination therapy but also mitigates the adverse effects associated with conventional tumor therapy. Finally, we outline the unresolved questions and suggest potential avenues for future investigations on cholesterol metabolism in relation to cancer.

## Introduction

The hallmarks of cancer are the acquired capabilities of cells during the transition from normal to neoplastic growth, facilitating the formation of malignant tumors. Six hallmarks of cancer were originally identified 1, but this number has since increased to fourteen. These include sustaining proliferative signaling, evading growth suppressors, resisting cell death, enabling replicative mortality, inducing or accessing vasculature, activating invasion and metastasis, deregulating cellular metabolism, avoiding immune destruction, genome instability and mutation, tumor-promoting inflammation, unlocking phenotypic plasticity, nonmutational epigenetic reprogramming, polymorphic microbiomes, and senescent cells 2. The tumor microenvironment (TME) contributes to the acquisition and maintenance of these hallmarks to varying degrees. The TME refers to a micro-ecosystem comprising non-cancerous cells and tumor components that provide nutritional support and growth-stimulating signals to tumor cells 3. The evolving understanding of TME has resulted in a shift in the focus of cancer therapy from a tumor-centric approach to a TME-centric approach. However, specialized microenvironments provide more precise targets for tumor treatment than the whole TME. The complex TME was previously divided into six specialized microenvironments 4. Recently, this classification has been expanded to include seven specialized microenvironments: hypoxic niche, immune microenvironment, metabolism microenvironment, acidic niche, innervated niche, mechanical microenvironment, and microbial microenvironment 2, 5. These specialized microenvironments interact with each other to form a dynamic tumor ecosystem.

Cholesterol is an essential constituent of the cell membrane and plays a vital role in cell survival and proliferation. In addition, cholesterol serves as a precursor for bile acids, steroid hormones, and oxysterol, which are crucial for maintaining various physiological processes 6. Therefore, the maintenance of cholesterol homeostasis is critical for physiological functions. Dysregulation of cholesterol homeostasis not only leads to cardiovascular diseases, but also involved in tumorigenesis and progression of cancer 7. The involvement of cholesterol in cancer has received increasing attention, with evidence of a dysregulated cholesterol balance in tumors. This imbalance in cholesterol homeostasis affects tumor hallmarks, promoting tumorigenesis, metastasis, and treatment resistance by reprogramming multiple microenvironments.

This review provides an overview of the regulatory mechanisms of cholesterol homeostasis and the effects of dysregulated cholesterol homeostasis on tumor hallmarks and interactions with various microenvironments. Finally, this paper provides a summary and discussion of the recent advances in the use of cholesterol metabolism as a target for cancer treatment.

## Brief overview of cholesterol homeostasis

**Figure 1:**
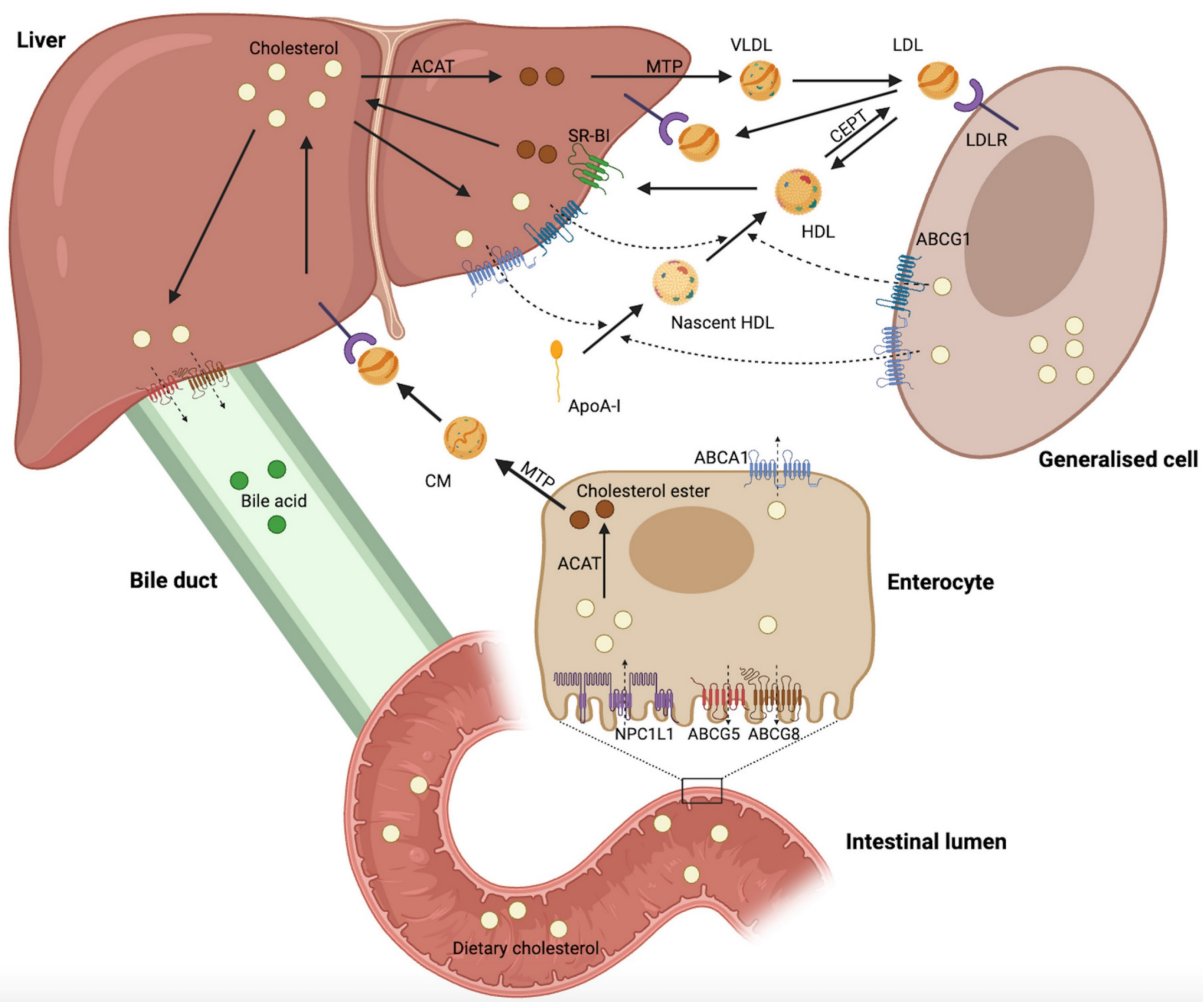
** Regulation of mammalian cholesterol homeostasis.** Hepatic cholesterol biosynthesis and dietary cholesterol primary sources of cholesterol in humans. Excess cholesterol in the liver is excreted into the bile and eventually into the intestinal lumen for fecal excretion. Cholesterol in the circulation can be excreted directly into the intestinal lumen via enterocytes. This figure was created using BioRender (https://biorender.com/).

**Figure 2:**
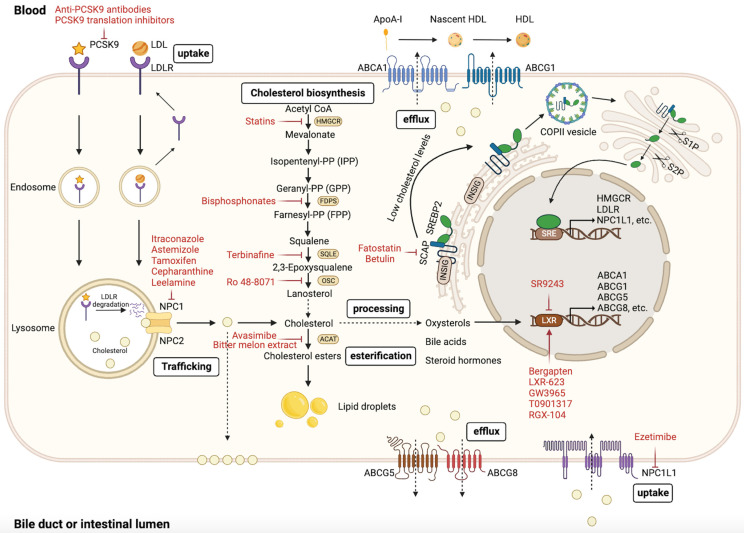
** Regulation of cellular cholesterol homeostasis.** The maintenance of cholesterol homeostasis is critical for physiological functions. Cellular cholesterol homeostasis is dynamically balanced through various processes, such as biosynthesis, uptake, esterification, efflux, and processing. Intracellular cholesterol levels are precisely regulated by these processes. This figure was created using BioRender (https://biorender.com/).

Cholesterol biosynthesis occurs in most mammalian cells, with hepatic cholesterol biosynthesis and dietary cholesterol being the primary sources of human cholesterol (Figure 1) 8. Generally, cholesterol homeostasis is dynamically maintained through various cellular processes, including biosynthesis, uptake, esterification, efflux, and processing (Figure 2). Cholesterol biosynthesis is accomplished through the mevalonate pathway, which not only provides a metabolic route of cholesterol synthesis, but also provides various metabolites with significant biological functions. The mevalonate pathway involves the conversion of acetyl-CoA, the end product of glycolysis, to 3-hydroxy-3-methyl-glutaryl-CoA (HMG-CoA), which is further metabolized through a series of enzymatic reactions to produce mevalonate, isopentenyl pyrophosphate (IPP), geranyl pyrophosphate (GPP), farnesyl pyrophosphate (FPP), squalene, lanosterol, and finally cholesterol 6. Cholesterol homeostasis is regulated by transcription factors, such as sterol regulatory element-binding protein 2 (SREBP2) and liver X receptor (LXR). SREBP2 monitors cholesterol levels in the endoplasmic reticulum (ER) and remains inactive until intracellular cholesterol levels decrease 9. Low cholesterol levels result in the release of the SREBP cleavage-activating protein (SCAP)-SREBP2 complex from the insulin-induced gene (INSIG) protein in the ER. This complex is then transported to the Golgi apparatus via COPII-coated vesicles, where SREBP2 undergoes proteolytic cleavage by site-1 (S1P) and site-2 (S2P) proteases 10. Upon cleavage, the N-terminus of SREBP2 enters the nucleus and activates the transcription of target genes, such as HMG‑CoA reductase (HMGCR), low-density lipoprotein (LDL) receptor (LDLR), and Niemann-Pick type C1-like 1 (NPC1L1). This results in elevated cholesterol biosynthesis and uptake 6, 10, 11. Elevated intracellular cholesterol levels result in an interaction between SCAP and INSIG proteins, preventing SCAP from binding to COPII and retaining the SCAP-SREBP2 complex within the ER, thereby impeding cholesterol biosynthesis. Furthermore, ER retention of the SCAP-SREBP2 complex modulates cholesterol uptake by reducing the expression of LDLR and NPCL1L 6. In addition to biosynthesis, diet and subsequent uptake of cholesterol from the circulation play a significant role in maintaining cholesterol homeostasis. Cells typically acquire cholesterol from circulation via LDLR-mediated endocytosis. LDLR binds to LDL in the bloodstream, and the resulting LDL-LDLR complex is transported to lysosomes for degradation. This process releases free cholesterol via Niemann-Pick C1 (NPC1) and C2 (NPC2) proteins 12. Proprotein convertase subtilisin/kexin type 9 (PCSK9) binds to LDLR and promotes its cellular uptake. Finally, the LDLR-PCSK9 complex undergoes lysosomal degradation 13. Dietary cholesterol is a major source of cholesterol in humans, and its uptake by enterocytes is mediated by the NPC1L1 protein 14. Following a series of processes, free cholesterol absorbed by NPC1L1 is esterified and transported as chylomicrons into circulation, ultimately being assimilated by the liver 14, 15. The human NPC1L1 gene comprises two sterol regulatory elements (SRE), the sterol-sensing structural domain, and is activated by SREBP2. A high-cholesterol diet was found to suppress NPC1L1 expression, suggesting negative feedback regulation between cholesterol content and its absorption pathway 16.

Maintaining cholesterol homeostasis requires ensuring sufficient cholesterol biosynthesis and uptake for cell growth and function, while also preventing the overabundance of intracellular cholesterol through esterification, efflux, and processing. When intracellular cholesterol levels exceed demand, excess cholesterol can be esterified to cholesterol esters (CEs) by acyl-CoA: cholesteryl acyltransferase (ACAT) and stored in the cytoplasm as lipid droplets (LDs) 17. In addition, cholesterol can be further metabolized into bile acids or steroid hormones secreted extracellularly 18, 19. Cholesterol serves as a vital precursor for oxysterols. Excess cholesterol can be converted into oxysterols, which are more polar and have distinct physiological functions 20. Activation of LXR by specific oxysterols increases the expression of cholesterol efflux-related genes, such as ATP-binding cassette (ABC) subfamily A member 1 (ABCA1), ABC subfamily G member 1 (ABCG1), ABCG5, and ABCG8 21. Excess cholesterol is exported into circulation via ABCA1 and ABCG1 and transported back to the liver as high-density lipoprotein (HDL) complexes 22, 23. ABCG5 and ABCG8 are expressed on the apical surface of hepatocytes and enterocytes, where they function as heterodimers to transport excess cholesterol to the bile duct and intestinal lumen 6.

The overview reveals that cholesterol homeostasis involves a complex regulatory network comprising various pathways and components, including cholesterol biosynthesis (HMGCR, SREBP2, SCAP, INSIG, S1P, and S2P), uptake (LDLR, PCSK9, and NPC1L1), esterification (ACAT), efflux (LXR, ABCA1, ABCG1, ABCG5, and ABCG8), and processing (bile acids, steroid hormones, and oxysterols). The complexity and accuracy of this regulatory network ensure a dynamic balance in intracellular cholesterol levels.

## Dysregulated cholesterol homeostasis as a contributor to the hallmarks of cancer

**Figure 3:**
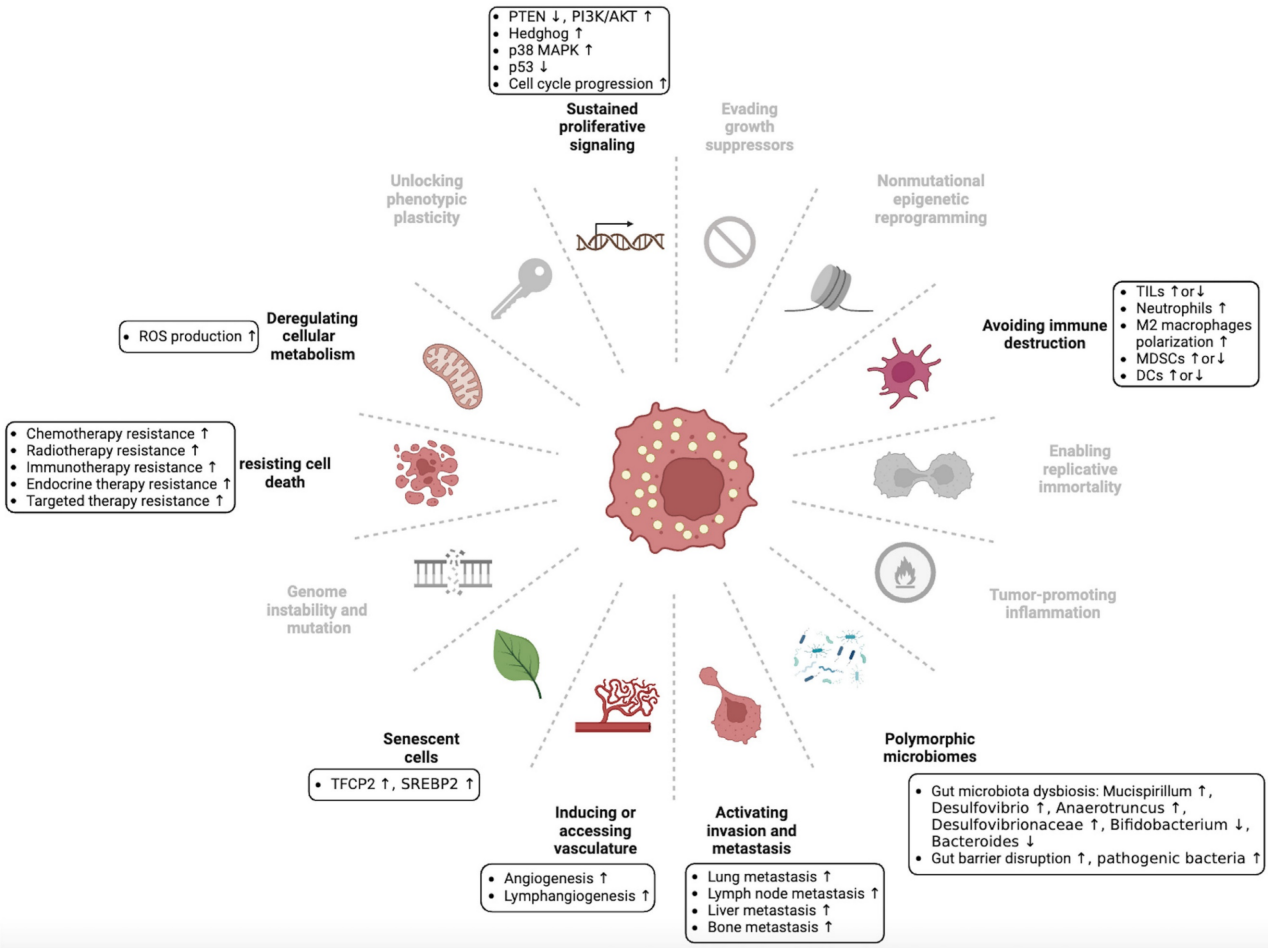
** Dysregulation of cholesterol homeostasis and hallmarks of cancer.** Hanahan and Weinberg originally proposed the concept of cancer hallmarks, which has since been expanded to encompass fourteen hallmarks. We summarize the association between dysregulated cholesterol homeostasis and some of these cancer hallmarks, although further exploration is necessary to fully understand this relationship. This figure was created using BioRender (https://biorender.com/).

Dysregulation of cholesterol homeostasis is a characteristic feature of cancer cells. Cancer cells require higher cholesterol levels for membrane formation and signal transduction because of their rapid proliferation compared with normal cells 24, 25. Lipid rafts, which contain cholesterol, are involved in diverse cellular processes 26. Lipid rafts are abundant in many cancer cells, and their disruption can inhibit cancer cell growth 27. Moreover, certain cholesterol precursors and derivatives have been found to affect cancer progression 28, 29. Recent studies have shown that dysregulated cholesterol homeostasis affects tumor development, progression, metastasis, and therapeutic resistance through various regulatory mechanisms (Figure 3 and Table 1) 30-33.

### Dysregulation of cholesterol homeostasis in the development and progression of cancer

The association between cholesterol and tumorigenesis has been explored since the last century. Several prospective cohort studies have indicated a positive correlation between high dietary cholesterol intake and elevated plasma cholesterol levels with cancer incidence 34, 35. Dysregulated cholesterol homeostasis is prevalent in many types of cancer and contributes to the onset and progression of the disease. Recent studies have reported various regulatory mechanisms by which dysregulation of cholesterol homeostasis affects cancer development and progression.

A recent report suggested that cholesterol and saturated fatty acids synergistically promote prostate cancer progression in mice 36. Wu et al. found that cholesterol activates the PI3K/AKT pathway to promote colorectal cancer (CRC) progression 37. In addition, PTEN loss and PI3K/AKT activation-induced cholesterol ester accumulation to promote prostate cancer progression 38. Cancer cells are characterized by their resistance to ferroptosis, a process closely linked to cholesterol biosynthesis. Liu et al. found that dysregulated cholesterol homeostasis contributes to ferroptosis resistance, promoting cancer tumorigenicity 7. *N*^1^-methyladenosine methylation in tRNA has been shown to drive liver tumorigenesis by inducing cholesterol biosynthesis, which activates Hedgehog signaling 39. SREBP2, a key transcription factor involved in cholesterol biosynthesis, is frequently upregulated in various cancers. Copy number amplification of gene alpha-endosulfine (ENSA) promotes triple-negative breast cancer (TNBC) progression by increasing SREBP2 expression 32. Gu et al. found that kinesin-like protein KIF11 promotes the progression of pancreatic ductal adenocarcinoma (PDAC) via SREBP2-dependent activation of mevalonate crosstalk 40. Furthermore, Wei et al. found that unspliced X-box binding protein 1 (XBP1) colocalizes with SREBP2 and inhibits its degradation, promoting cholesterol biosynthesis and hepatocellular carcinoma (HCC) tumorigenesis 41. Cellular senescence is associated with suppression of tumorigenesis 42-44, and tumor cells use various strategies to overcome senescence. A recent study found that transcription factor CP2 (TFCP2) interacts with SREBP2 to synergistically activate cholesterol biosynthesis and overcome cellular senescence in pancreatic cancer 45. Oxysterols, being a derivative of cholesterol, have been extensively studied for their association with cancer due to their beneficial and detrimental effects on the disease 46, 47. CYP27A1, a cytochrome P450 oxidase, catalyzes the conversion of cholesterol to 27-hydroxycholesterol (27HC) 48. CYP27A1 has demonstrated renal cell carcinoma (RCC)-inhibiting effects by increasing 27HC concentrations in the body 49. Similarly, Liang et al. found that CYP27A1 activates LXRs/ABCA1 by upregulating 27HC, promoting intracellular cholesterol efflux, and eventually inhibiting proliferation and migration in clear cell renal cell carcinoma (ccRCC) 50. Evidence suggests that 27HC may promote the onset and progression of tumors. Luo et al. found that the histone reader zinc finger MYND-type containing 8 (ZMYND8) activates LXR and promotes breast cancer initiation by inhibiting 27HC catabolism and cholesterol efflux 51. Avena et al. reported that 27HC promotes the progression of estrogen receptor-negative breast cancer (ER-BC) by binding to G protein-coupled estrogen receptor (GPER) 52. In addition, 22HC induces cell cycle arrest by activating LXR, thereby inhibiting the progression of prostate, breast, and liver cancers 53. However, Yoon et al. found that 22HC contributes to the development and progression of cholangiocarcinoma by stimulating COX-2 expression via a p38 MAPK-dependent mechanism 54. Other oxysterols, such as 24HC and 25HC, have been identified as significant contributors to the development and progression of cancer 55-58. PCSK9 promotes LDLR degradation, leading to increased circulating LDL levels. PCSK9 is a key regulator of cholesterol homeostasis and has been implicated in various aspects of cancer biology 59. PCSK9 overexpression in CRC promotes cholesterol biosynthesis and accumulation of its intermediate geranylgeranyl diphosphate (GGPP) by inhibiting cholesterol uptake, ultimately inducing tumorigenesis 60. In addition, squalene epoxidase (SQLE), the rate-limiting enzyme of cholesterol biosynthesis, has also been shown to promote the progression of p53-deficient castration-resistant prostate cancer (CRPC) 61, proliferation of p53 wild-type HCC cells, liver tumorigenesis in p53 knockout mice 62, and colorectal carcinogenesis by promoting gut dysbiosis 63. Interestingly, Zhang et al. also found that dietary cholesterol drives the development of non-alcoholic fatty liver disease in HCC by altering gut microbiota and metabolites 64. These findings establish a correlation between cholesterol homeostasis, gut microbiota, and tumorigenesis, providing a novel approach to exploring the involvement of cholesterol homeostasis in tumorigenesis.

Clinical and preclinical studies have demonstrated a strong link between cholesterol homeostasis and cancer. However, the mechanism by which dysregulated cholesterol homeostasis contributes to the onset and progression of cancer remains to be fully elucidated.

### Dysregulation of cholesterol homeostasis in tumor metastasis

Cancer metastasis is a dynamic and multi-step process 65, 66. The prognosis of patients with metastatic cancer is extremely poor, with over 90% of cancer-related deaths attributable to metastasis 67. However, the regulatory mechanisms that underlie cancer metastasis remain unclear. The correlation between cholesterol homeostasis and the mechanism of cancer metastasis is currently under investigation.

A clinical study highlighted that pretreatment serum cholesterol levels were higher in patients with metastatic prostate cancer than those with nonmetastatic prostate cancer 68. Additionally, Liu et al. found that dysregulated cholesterol homeostasis in breast cancer promotes metastasis by inducing resistance to ferroptosis 7. Han et al. revealed that regulation of the cholesterol synthesis pathway through a chemokine regulatory loop could promote metastatic growth of lung-colonizing TNBC cells 30. Kim et al. reported that the cholesterol synthesis pathway increases tumor sphere formation and invasion in breast cancer cell metastasis 69. Chen et al. found that the inhibition of LDLR expression in liver cancer stimulates intracellular cholesterol biosynthesis via the MEK/ERK signaling pathway, thereby promoting lung metastasis of HCC 70. Interestingly, PCSK9, an inhibitor of LDLR, inhibits the lung metastasis of HCC 71, but stimulates lung metastasis of melanoma cells 72. Xu et al. reported that PCSK9 could enhance lung and lymph node metastasis in gastric cancer (GC) by upregulating heat shock protein 70 levels and promoting the MAPK signaling pathways 73. Sun et al. found that PCSK9 deficiency reduced liver metastasis in melanoma by decreasing cholesterol levels 74. Oxysterols have also been implicated in cancer metastasis. Ortiz et al. found that cholesterol 25-hydroxylase (CH25H) produces 25HC, which inhibits the uptake of tumor-derived extracellular vesicles by normal cells and restricts the development of premetastatic niches in melanoma 75. 25HC promotes lung metastasis of gastric cancer by upregulating TLR2/NF-κB mediated matrix metalloproteinase (MMP) expression 76. Additionally, Nelson et al. found that 27HC promotes breast cancer lung metastasis by activating LXR 77. Baek et al. reported that 27HC promotes the distant metastasis of breast cancer cells to the lungs by interacting with γδ-T cells and polymorphonuclear neutrophils 78. Recently, Deng et al. reported that anoctamin 1 (ANO1) interacts with JUN to suppress CYP27A1-LXR signaling, leading to intracellular cholesterol accumulation and TME reprogramming, thus enhancing esophageal squamous cell carcinoma metastasis 79. Late-stage lung cancer is associated with a higher incidence of osteolytic bone metastases 80. Zhang et al. found that 27HC promotes the colonization of lung adenocarcinoma cells in the bone by stimulating osteoclast differentiation and creating a favorable microenvironment for tumor growth 81. In addition, elevated cholesterol levels have been observed in bone metastases from prostate cancer 82. SREBP2 facilitates distant metastasis of prostate cancer to the bone, adrenal gland, and lungs by transcriptionally activating c-Myc 83 and promotes bone metastasis in breast cancer by regulating osteoclast formation and function 84. When intracellular cholesterol levels are high, sterol O-acyltransferase 1 (SOAT1), also known as ACAT1, converts excess cholesterol to cholesteryl ester (CE) 9. SOAT1 is overexpressed in cancer and associated with poor patient prognosis 47, 85, 86. Studies have shown that SOAT1 promotes lymph node metastasis in GC by regulating the expression of SREBP1 and SREBP2 87, and its inhibition significantly suppresses lymph node and liver metastasis of pancreatic cancer 88. Furthermore, Du et al. revealed that fatty acid synthase promotes cervical cancer lymph node metastasis by regulating cholesterol reprogramming and inducing lymphangiogenesis in cervical cancer 89.

Therefore, dysregulation of cholesterol homeostasis can contribute to cancer metastasis through multiple mechanisms. Consequently, there has been extensive research on potential drugs that target cholesterol metabolism for cancer therapy. Statins, a well-studied class of repurposed drugs that target cholesterol biosynthesis, have emerged as promising anticancer agents because of their ability to inhibit cancer metastasis via multiple mechanisms 90. However, further investigation is required to understand the mechanism by which dysregulated cholesterol homeostasis promotes cancer metastasis, and to develop effective anticancer drugs targeting cholesterol metabolism.

### Dysregulation of cholesterol homeostasis promotes cancer therapeutic resistance

Advancements in cancer research have led to significant progress in the exploration and implementation of various anticancer strategies. However, therapeutic resistance often results in treatment failure in most cancer patients. Studies have shown that dysregulated cholesterol homeostasis contributes significantly to resistance against cancer therapeutic strategies, including chemotherapy, radiotherapy, immunotherapy, endocrine therapy, and targeted therapy through multiple mechanisms.

Chemotherapy is a common treatment for cancer, and the dysregulation of cholesterol homeostasis has been linked to chemotherapy resistance in various studies. In pancreatic ductal adenocarcinoma, Yu et al. found that overexpression of cellular retinoic acid-binding protein II (CRABP-II) induces cholesterol accumulation in lipid rafts by upregulating the downstream SREBP-1c and eventually promoting resistance to gemcitabine 91. Similarly, 27HC promotes the proliferation of prostate cancer cells and induces resistance to docetaxel via an androgen receptor (AR)-dependent mechanism 92. Furthermore, Wang et al. demonstrated that 25HC promotes 5‑fluorouracil (5-FU) resistance in human GC cells 76. Autophagy plays an important role in cell growth and development and has a biphasic effect on cancer progression, with studies demonstrating its ability to both suppress and promote tumors at different stages 93. Lipid raft deficiency has been linked to doxorubicin resistance in breast cancer by promoting autophagy 94, whereas activation of the mevalonate pathway has been associated with doxorubicin resistance in bladder cancer cells 95. Cholesterol and HOXB13 have been found to induce resistance to platinum-based chemotherapy in lung adenocarcinoma by upregulating ABCG2 and ABCG1 expression, respectively 96, 97. The SREBP2 pathway 98 and malignant ascites cholesterol have been found to contribute to cisplatin resistance in ovarian cancer cells. Malignant ascites cholesterol activates LXRɑ/β, which increases the resistance of ovarian cancer cells to cisplatin and paclitaxel 99. Research indicates that inhibition of PCSK9 can protect prostate cancer cells from radiation-induced cell damage, suggesting that PCSK9 may be a promising therapeutic target for enhancing radiosensitivity in prostate cancer 100. Programmed death ligand 1 (PD-L1) is a therapeutic target in cancer immunotherapy, and PD-L1 inhibitors have been successfully used to treat cancer by restoring T-cell tumor-killing activity 101, 102. However, tumor cell resistance to PD-L1 inhibitors is a significant concern. Studies have shown that statin treatment significantly lowers the expression of PD-L1, suggesting that cholesterol is closely related to PD-L1 103. Recently, Wang et al. found that the transmembrane domain of PD-L1 contains two cholesterol-recognition amino acid consensus (CRAC) motifs that can be recognized and bound by cholesterol, resulting in increased stability of PD-L1 in cancer cells and immunoevasion 104. Furthermore, although endocrine therapy has been proven beneficial for numerous patients, the emergence of resistance to endocrine therapy has become a growing concern in recent years. Research indicates that 25HC may induce resistance to estrogen deprivation in estrogen receptor-positive (ER+) breast cancer by mimicking hormones 105. Recently, Palma et al. reported that cholesterol depletion sensitizes breast cancer cells to tamoxifen 106. HMGCR is a crucial enzyme in cholesterol biosynthesis. Kong et al. found that HMGCR overexpression promoted resistance of CRPC to enzalutamide, and statins were effective in overcoming this resistance 107. Furthermore, El-Kenawi et al. found that cholesterol-rich macrophages induce resistance to endocrine therapy in CRPC by transferring cholesterol to cancer cells 108.

Targeted therapy is a type of precision medicine that provides optimism for cancer treatment. The continuous emergence of targeted drugs has significantly improved the prognosis of cancer patients. However, drug resistance presents a significant challenge for cancer treatment. Therefore, exploring the resistance mechanisms of targeted drugs can offer insight into improving their therapeutic efficacy. Cholesterol induces resistance to epidermal growth factor receptor tyrosine kinase inhibitors (EGFR-TKIs) in non-small cell lung cancer (NSCLC) via the EGFR/Src/Erk/SP1 signaling pathway 31, while elevated levels in lipid rafts induce gefitinib resistance 109. SCAP and caspase-3 promote HCC resistance to sorafenib via AMPK-mediated autophagy 110 and SREBP2-induced sonic hedgehog signaling 111, respectively. Interestingly, statins can overcome sorafenib resistance in HCC 111 and delay GC resistance to Trastuzumab 112.

Cancer therapeutic resistance is a major concern that requires further investigation. Studies have demonstrated a correlation between cholesterol homeostasis and therapeutic resistance, with ongoing research aimed at understanding the underlying mechanisms of cholesterol-induced therapeutic resistance. Fortunately, cholesterol biosynthesis-targeting drugs have shown significant efficacy in overcoming therapeutic resistance. In the future, the combination of cholesterol metabolism-targeting drugs with conventional anticancer drugs may serve as a promising novel cancer therapeutic strategy.

## Cholesterol metabolism and the TME

**Figure 4:**
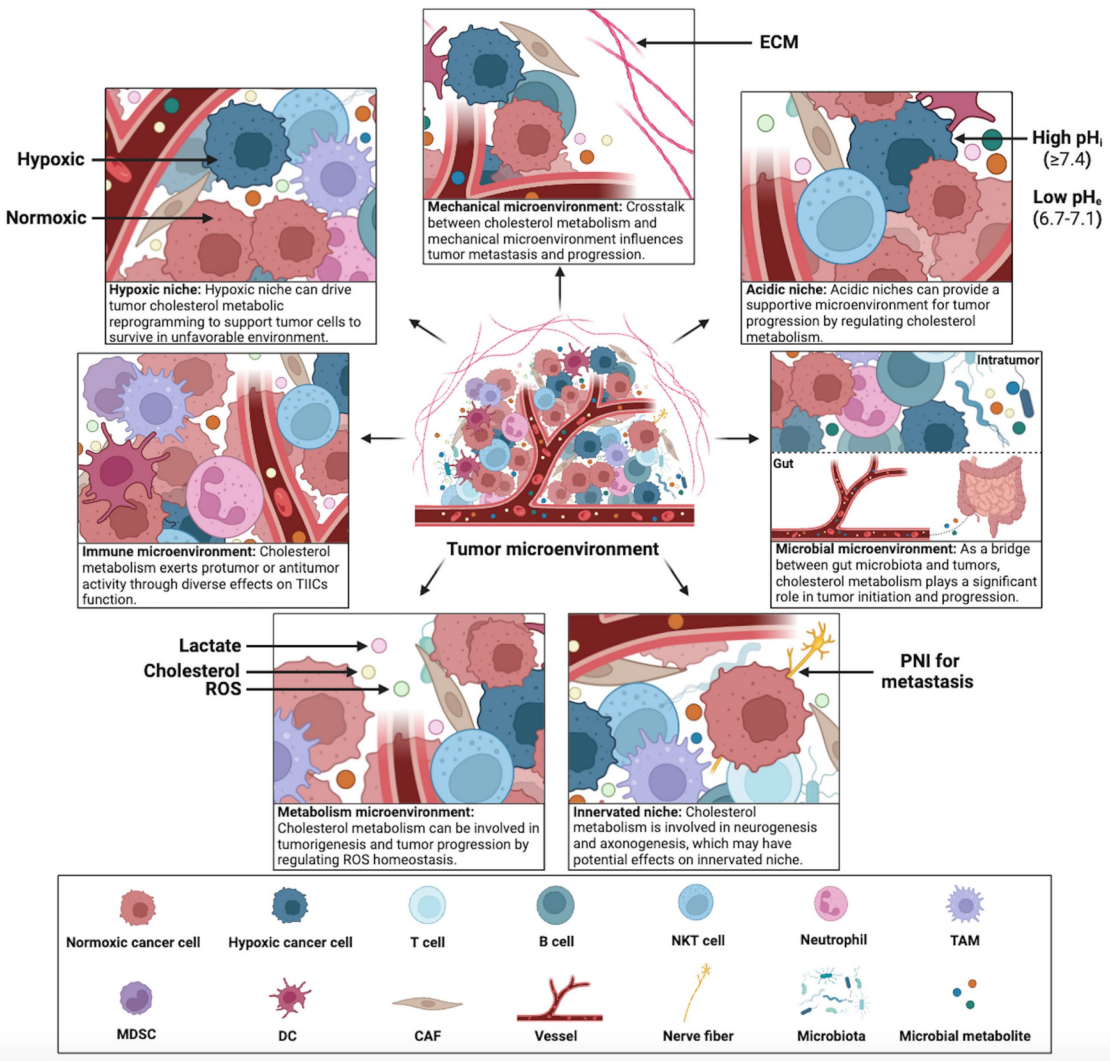
** Crosstalk between cholesterol metabolism and the TME.** The TME has been categorized into seven specialized microenvironments: hypoxic niche, immune microenvironment, metabolism microenvironment, acidic niche, innervated niche, mechanical microenvironment, and microbial microenvironment (including intratumor and gut microbiota). Crosstalk between cholesterol metabolism and specialized microenvironments plays a significant role in tumorigenesis and tumor progression. This figure was created using BioRender (https://biorender.com/).

Constant interaction between tumor cells and their microenvironment is a crucial factor in the development, progression, metastasis, and therapeutic outcomes of cancer. Cholesterol metabolism appears to contribute significantly to this interaction, tumor cells in the TME can adapt to the complex microenvironment by reprogramming cholesterol metabolism. Therefore, exploring the mechanisms of cholesterol metabolism in seven specialized microenvironments can provide insights into the crosstalk between tumor cells and the complex TME. This section summarizes the diverse effects of cholesterol metabolism on hypoxic niche, immune microenvironment, metabolism microenvironment, acidic niche, innervated niche, mechanical microenvironment, and microbial microenvironment (Figure 4).

### Cholesterol metabolism and immune microenvironment

During all stages of tumor progression, there is a constant interplay between tumor cells and tumor-infiltrating immune cells (TIICs) in the tumor immune microenvironment (TIME). These TIICs comprise immune effector and immunosuppressive cells, including T lymphocytes, B lymphocytes, natural killer (NK) cells, neutrophils, tumor-associated macrophages (TAMs), myeloid-derived suppressor cells (MDSCs), and dendritic cells (DCs), which perform diverse antitumor or protumor functions in the TME 113, 114. Evidence suggests that cholesterol metabolism influences antitumor immunity by acting on various TIICs 115.

Tumor-infiltrating lymphocytes (TILs) recognize and kill tumor cells, and cytotoxic T lymphocytes (CTLs) play a central role in antitumor immunity. Cholesterol metabolic reprogramming occurs in the TME during the functional maturation and activation of TILs. Cholesterol is required for T cell proliferation and activation. SREBP-mediated upregulation of cholesterol biosynthesis plays an important role in the activation and proliferation of CD8^+^ T cells 116. Research has demonstrated that inhibiting ACAT1 activity upregulates plasma membrane cholesterol levels in CD8^+^ T cells, which results in enhanced T cell receptor clustering and signal transduction, as well as more efficient immunological synapse formation and antitumor response 117. Wang et al. recently found that SOAT1-targeting compounds reprogrammed cholesterol metabolism in tumor cells and enhanced the antitumor response of CD8^+^ T cells against liver cancer 118. Additionally, trogocytosis has been identified as an important process in immune regulation and other biological processes 119. Trogocytosis refers to the extraction and transfer of biomolecules between adjacent cells, resulting in changes in both donor and acceptor cell functions 119, 120. Trogocytosis between tumor cells and CTLs promotes the loss of antigens on target cells and destruction of CTLs, enabling tumor cells to evade the immune system 121. Recent studies have shown that CH25H can enhance antitumor immunity by inhibiting trogocytosis and stimulating CTL activity 122. Furthermore, another study found that CH25H promotes major histocompatibility complex class I (MHC-I) presentation and increases CD8^+^ T cell infiltration into tumors, sensitizing PDAC cells to immune checkpoint inhibitors 123. In contrast, 27HC promotes the development of the breast cancer premetastatic niche by attracting polymorphonuclear neutrophils and γδ-T cells at metastatic sites while depleting CD8^+^ T cells 78. Recently, Yan et al. found that 27HC induces cholesterol deficiency in T cells by inhibiting SREBP2 and activating LXR, subsequently leading to autophagy-mediated apoptosis of T cells 124.

Interestingly, increasing cholesterol levels of chimeric antigen receptor (CAR)-T cells by blocking LXR can enhance antitumor activity, suggesting that improving CAR-T therapy by regulating cholesterol metabolism is a promising antitumor strategy 124. Cytotoxic NK cells, which are crucial for antitumor immunity, are also affected by cholesterol metabolism. Qin et al. found that upregulating LDLR expression in NK cells improved their ability to combat HCC by elevating intracellular cholesterol levels, suggesting that increasing cholesterol uptake in NK cells could be a promising therapeutic strategy for HCC 125. Yuan et al. found that the antitumor activity of CD8^+^ T cells is enhanced by LDLR via regulation of T-cell receptor (TCR) cycling and signaling through its interaction with the TCR complex 126 and inhibited by PCSK9 via inhibition of the recycling of LDLR and TCR to the CD8^+^ T cell plasma membrane 126. Furthermore, Liu et al. revealed that PCSK9 binds to MHC-I in lysosomes and triggers its degradation, thereby preventing CTL infiltration and inhibiting antitumor immunity 127. These findings suggest that PCSK9 inhibition could be a promising strategy for enhancing the immune response against tumors. However, the effect of cholesterol on T cell function is controversial, and high cholesterol levels in the TME have been linked to CD8^+^ T cell exhaustion by triggering immune checkpoint activation and ER stress 128. Therefore, reducing cholesterol levels in the TME can effectively restore the antitumor activity of CD8^+^ T cells 128. CD8^+^ T cell subset interleukin-9 (IL-9)-secreting (Tc9) cells have stronger antitumor activity than Tc1 cells, and IL-9 is indispensable for the antitumor activity of Tc9 cells. Cholesterol suppresses IL-9 expression by activating LXRs, thereby inhibiting the antitumor activity of Tc9 cells 129. Recent studies have shown that colorectal cancer with microsatellite stability (MSS CRC) cells polarize T cells toward Th17 cells by secreting distal cholesterol precursors, thus promoting tumor progression 130. SREBP2-mediated hepatic cholesterol accumulation suppresses natural killer T (NKT) cell cytotoxicity and antitumor immunosurveillance in liver cancer 131. Wang et al. found that reducing serum cholesterol levels inhibited the mammalian target of rapamycin complex 2 (mTORC2) signaling in T lymphocytes and increased CD8^+^ T cell infiltration in prostate cancer 132. These findings suggest that intrinsic and extrinsic cholesterol may exert differential effects on the function of TILs. Increasing intrinsic cholesterol biosynthesis and uptake of TILs may enhance their antitumor activity, whereas excessive extrinsic cholesterol in the TME may inhibit it. Moreover, B cells play a crucial role in the regulation of immune responses to tumors. Recent research indicates that SREBP signaling is crucial for B cell maturation, suggesting the significance of cholesterol metabolism in B cell regulation 133. Bibby et al. found that cholesterol metabolism promotes the production of IL-10 from regulatory B cells via GGPP, thus restraining the immune response 134.

In addition to TILs, other TIICs have also been shown to be regulated by cholesterol metabolism. In the TIME, neutrophils typically exhibit protumor activity, and cholesterol derivatives, oxysterols, have been reported to exert protumor activity by regulating neutrophils. Raccosta et al. found that tumor-derived 22HC can recruit neutrophils via CXC chemokine receptor 2 (CXCR2) and promote tumor growth by stimulating angiogenesis and immunosuppression 135. Soncini et al. found that hypoxia-inducible factor-1α (HIF-1α) can enhance neoangiogenesis in pancreatic neuroendocrine tumors by attracting neutrophils via 24HC upregulation 55. Furthermore, 27HC has been reported to promote breast cancer metastasis through the recruitment of polymorphonuclear neutrophils at metastatic sites 78. Tumor-infiltrating myeloid cells, including TAMs, MDSCs, and DCs, are also important components of the TIME. Cholesterol metabolic reprogramming can shift these myeloid cells towards an antitumor or a protumor phenotype. TAMs exhibit distinct antitumor M1-like and protumor M2-like phenotypes. Studies have suggested that tumors can alter cholesterol metabolism to promote the protumor function of TAMs. For instance, ovarian cancer cells secrete hyaluronic acid to promote cholesterol efflux from TAM membranes and induce a protumor M2-like phenotype 136. Moreover, glioblastoma multiforme (GBM)-derived 25HC can activate the recruitment of macrophages to tumors through the G-protein-coupled receptor 183 (GPR183) 137. MDSCs, as immature myeloid cells, typically exhibit immunosuppressive properties in TIME. Recently, Yang et al. reported that the UPR component XBP1 stimulates cholesterol biosynthesis and secretion in tumor cells, activating MDSCs to exhibit immunosuppressive properties 138. In contrast, Tavazoie et al. found that LXRβ reduces the infiltration of protumor MDSCs into tumors, thus enhancing antitumor immunity 139. However, Xie et al. recently found that chronic activation of LXRα can have an immunosuppressive effect by increasing the infiltration of MDSCs into tumors 140. Therefore, future studies must elucidate the contradictory functions of LXRs in the immune response to cancer. The maturation of antigen-presenting DCs is essential for the activation and maintenance of the antitumor activity of TILs. Zhang et al. found that cholesterol-modified antimicrobial peptide DP7 induces DCs maturation and enhances antigen presentation by transporting various antigen peptides into DCs 141. Xia et al. found that inhibiting geranylgeranylation of Rab5 in DCs resulted in cell surface antigen retention, enhanced antigen presentation, and CD8^+^ T cell activation 142. Villablanca et al. found that oxysterol in the TME inhibits CC chemokine receptor-7 (CCR7) expression on the surface of mature DCs by activating LXRα, thereby inhibiting DC migration to lymphoid organs and reducing antitumor immunity 143. In contrast, another study showed that LXR inverse agonists facilitate DC migration to the lymph nodes and boost antitumor immunity 144. These findings suggest that the inhibition of LXR expression may be a promising immunotherapeutic approach for tumors.

In summary, cholesterol metabolism is critical for regulating the crosstalk between tumor cells and TIME. Cholesterol metabolism affects TIICs in the TIME through multiple mechanisms, resulting in either protumor or antitumor effects on the function of TIICs. Therefore, a deeper understanding of the regulation of cholesterol metabolism in the interaction between tumor cells and TIME will facilitate the development of novel immunotherapeutic strategies for cancer treatment. Targeting cholesterol metabolism in TIME has the potential as an effective antitumor approach by augmenting immunity against tumors.

### Cholesterol metabolism and metabolism microenvironment

Metabolic reprogramming is a fundamental characteristic of cancer cells. Aerobic glycolysis (Warburg effect) is an early indication of metabolic reprogramming in cancer, that is, cancer cells prefer glycolysis to oxidative phosphorylation even under normoxic conditions 145. Aerobic glycolysis allows cancer cells to compete with surrounding normal cells for glucose uptake, thereby sustaining their growth 146. Lactate, produced through aerobic glycolysis, has significant effects on TME and tumor-associated cells 147. Aerobic glycolysis can mitigate excessive accumulation of reactive oxygen species (ROS) by avoiding oxidative phosphorylation 148. ROS plays a dual role in tumorigenesis by promoting tumor onset and progression via activation of various redox reactions and signaling pathways while also causing tumor cell damage and eventual death through oxidative stress 149. Tumor cells exhibit higher levels of ROS than normal cells, and continuous oxidative stress causes them to tolerate slight accumulation of ROS (known as ROS addiction) 149. Increasing evidence suggests that ROS addiction contributes to tumorigenesis and tumor progression. Cholesterol metabolism might play a role in this process by regulating ROS homeostasis.

Cholesterol accumulation in the tumor cell mitochondria triggers a cascade of chain reactions, leading to ROS production 150. Interestingly, in ovarian cancer, increased mitochondrial ROS generation and activation of the AKT/mTOR signaling pathway upregulate SREBP2 expression, thereby promoting cholesterol biosynthesis 151. Furthermore, increased cholesterol uptake protects against ROS-induced damage in ccRCC 152. Shapira et al. found that cholesterol depletion decreased autophagic flux in an ROS- and JNK-dependent manner 153. Wang et al. found that cholesterol promotes CRC progression by activating ROS and MAPK signaling pathway 154. Liu et al. demonstrated that SQLE contributes to HCC tumorigenesis by silencing PTEN via ROS-induced DNA methyltransferase 3A (DNMT3A) expression 155. In addition, the interaction between 27HC and ROS has been linked to both drug resistance and tumor progression. 27HC activates glucose-regulated protein 75 (GRP75) via elevated oxidative stress signaling in HCC, which regulates redox homeostasis by regulating ROS production and the antioxidant defense system, thereby inducing multidrug resistance 156. Furthermore, evidence suggests that ROS contributes to 27HC-induced breast cancer cell invasion and angiogenesis by regulating reversion-inducing-cysteine-rich protein with Kazal motifs (RECK)/STAT-3 signaling via DNA methylation 157 and STAT-3/VEGF signaling 158, respectively.

### Cholesterol metabolism and hypoxic niche

Tumors exhibit high adaptability, which enables them to thrive under adverse conditions. Intratumoral hypoxia is induced by rapid tumor growth and inadequate angiogenesis. Hypoxia-inducible factors (HIFs) are significant regulators of hypoxic response 159. Hypoxia activates vascular endothelial cells, stimulates tumor angiogenesis, and promotes tumor growth and metastasis 160, 161. More importantly, the hypoxic response triggers the “angiogenic switch” and promotes metabolic reprogramming in tumors 66, 162. Evidence suggests that tumors can adapt to these hypoxic environments by reprogramming cholesterol metabolism to enhance tumor stemness and angiogenesis, which promotes their survival and growth. HIF-1α facilitates SREBP maturation in the hypoxic niche by upregulating the Ephrin-A3/EphA2 axis expression, thus enhancing cancer stemness in HCC 163. Hypoxia activates HIF-1 by inducing protein kinase B (PKB) phosphorylation, thereby upregulating SREBP expression in breast cancer cells 164. In addition, HIF-1α upregulates 24HC to attract neutrophils to hypoxic areas and induce the occurrence of “angiogenic switch” in pancreatic neuroendocrine tumor 55.

In summary, the hypoxic niche drives cholesterol metabolic reprogramming in tumors and interacts with other specialized microenvironments, promoting the survival of tumor cells under unfavorable conditions.

### Cholesterol metabolism and acidic niche

Cancer is characterized by dysregulated or reversed pH, with a higher intracellular pH (pH_i_ ≥ 7.4) and a lower extracellular pH (pH_e_ = 6.7-7.1) 165. Higher pH_i_ facilitates cancer cell proliferation, migration, metabolic adaptation, and anti-apoptosis, whereas lower pH_e_ facilitates cancer cell invasion and metastasis 165. The acidic niche is closely linked to the hypoxic niche and metabolism microenvironment because the acidic niche is primarily caused by lactate secretion and CO_2_ production from anaerobic glycolysis and the pentose phosphate pathway, respectively 166. Evidence suggests that an acidic niche provides a supportive microenvironment for tumor progression by regulating cholesterol metabolism.

Acidic extracellular environments can promote cholesterol biosynthesis in tumor cells by activating SREBP2, thereby promoting tumor growth 167. Importantly, acidic pH-regulated SREBP2 target genes are inversely correlated with the overall survival of cancer patients 167. Fukamachi et al. found that reducing GGPP synthesis can inhibit the proliferation of synovial sarcoma cells in acidic environments 168. Additionally, John et al. found that activation of the IRE1-sXBP1-SREBP2-ACSS2 response axis at low pH_e_ promotes cholesterol biosynthesis and cell membrane surface trafficking in astrocytic tumors, thereby enhancing cell membrane surface mechanical tenacity, preventing acid-mediated cell membrane hydrolysis, and supporting tumor cell survival 169. In addition, Corbet et al. found that acidosis-induced TGF-β2 activation facilitated LD accumulation, enhancing the distant metastatic potential of cancer cells 170.

### Cholesterol metabolism and mechanical microenvironment

The mechanical microenvironment, which is comprised of intracellular components (neurofilaments, vimentin, and actin), extracellular components (fibrin and collagen), stromal cells (fibroblasts), and intercellular signaling (integrin and focal adhesion) has been shown to affect intracellular signal transduction and, consequently, the biological behavior of tumor cells 171, 172. Cholesterol metabolism appears to play a significant role in the mechanical microenvironment.

Cancer-associated fibroblasts (CAFs) are primary stromal cells in the mechanical microenvironment of tumors and can contribute to metastasis by secreting MMPs or activating yes-associated protein (YAP), leading to extracellular matrix (ECM) remodeling and epithelial-mesenchymal transition (EMT) 173-175. Han et al. observed that TNBC-derived C-X-C motif chemokines 1/2/8 (CXCL1/2/8) stimulate CAFs and other lung-resident fibroblasts to secrete C-C motif chemokines 2/7 (CCL2/7), thereby activating cholesterol synthesis in TNBC cells to support metastatic tumor growth 30. Moreover, 25HC was found to promote lung metastasis of GC by upregulating MMP expression 76. Enhanced mevalonate pathway signaling by mutant p53 promotes the activity of YAP and PDZ-binding motif (TAZ) proto-oncogenes 176, whereas the oncogenic activity of YAP is dependent on ZMYND8-mediated cholesterol biosynthesis 177. Cholesterol is essential for receptor signaling (e.g., integrin) by maintaining the stability of lipid rafts. Ramprasad et al. reported that depletion of cholesterol in the plasma membrane decreases α5β1 integrin-mediated adhesion and motility of lung adenocarcinoma cells to fibronectin 178. In addition, Hoque et al. found that LDL-cholesterol promotes the motility and spread of tumor cells by participating in integrin trafficking, focal adhesion assembly, and ECM secretion 179. Recently, transcription factor EB (TFEB) was shown to facilitate integrin-mediated endothelial cell adhesion to the ECM by upregulating cholesterol synthesis-associated genes in response to integrin signaling 180. Maja et al. found that cholesterol-enriched cell surface domains are involved in ECM degradation, which promotes breast cancer cell invasion 181. In addition, Shen et al. found that 27HC increases MMP9 expression and EMT through STAT-3 activation, thus promoting breast cancer migration and invasion 182. This effect has also been validated by Avena et al. and Torres et al., who demonstrated that 27HC promotes EMT and migration of breast cancer cells 52, 183. High cholesterol-mediated upregulation of adipocyte plasma membrane-associated protein (APMAP) in cholesterol-induced lipid rafts inhibits EGFR degradation, thereby activating the extracellular-regulated protein kinase 1/2 (ERK1/2) pathway and inducing EMT in prostate cancer cells 184. Therefore, crosstalk between cholesterol metabolism and the mechanical microenvironment influences tumor metastasis and progression.

### Cholesterol metabolism and microbial microenvironment

The microbial microenvironment is an emerging specialized microenvironment that delineates a landscape composed of intratumor microbiota, intestinal microbiota, and their metabolites 5, 185. Intestinal microbiota plays an indispensable role in cholesterol metabolism. In this section, we discuss the interplay between cholesterol metabolism and the microbiota in the initiation and progression of tumors.

Zhang et al. found that dietary cholesterol contributes to non-alcoholic fatty liver disease (NAFLD)-associated HCC formation by inducing gut microbial dysbiosis 64, which could be effectively prevented by statin treatment 64. This suggests a potential association between changes in the gut microbiota and tumorigenesis. Additionally, Li et al. proposed that SQLE promotes CRC carcinogenesis by modulating the gut microbiota-metabolite axis 63. Cholesterol is converted to primary bile acids in the liver and subsequently processed into secondary bile acids by the gut microbiota 186. Accumulating evidence suggests that secondary bile acids can promote tumorigenesis 187-190. Yamada et al. found that the accumulation of secondary bile acids produced by the gut microbiota can activate mTOR signaling pathways in hepatocytes, thus promoting HCC carcinogenesis 191. Primary bile acids have a positive effect on antitumor immunity by increasing CXCL16 expression in liver sinusoidal endothelial cells, consequently promoting the accumulation of CXCR6^+^ hepatic NKT cells 192. In contrast, gut microbiome-mediated secondary bile acids suppress antitumor immunity by inhibiting the accumulation of hepatic NKT cells 192. This suggests that cholesterol metabolism plays a significant role in the microbial microenvironment-TIME crosstalk.

In summary, cholesterol metabolism serves as a bridge between the intestinal microbiota and tumors and plays a significant role in the initiation and progression of tumors by regulating intestinal homeostasis and the immune system.

### Cholesterol metabolism and innervated niche

With increasing awareness of the interaction between neurology and cancer science, Monje et al. have proposed a new field of study, “cancer neuroscience,” to explore bidirectional interactions between the nervous system and cancer 193. We propose that the crosstalk between nerves and cancer is mediated by acellular components such as nerve-derived neurotransmitters or neuropeptides, resulting in a specialized microenvironment called “innervated niche” 4. Some previous studies have also used the terms “perineural niche,” “neural regulation in TME,” or “neural microenvironment” 194-196.

Currently, there is a lack of evidence to support the effect of cholesterol metabolism on innervated niches despite the significant role of cholesterol in the nervous system 197. Cholesterol is a vital structural component of neuronal plasma membranes and is necessary for maintaining fluidity and proper functioning of neurons 198. Myelin is the continuous extension of the neuronal plasma membranes, with a higher lipid content, including 25-30% cholesterol 199. Cholesterol is also involved in synapse and dendrite formation 200, and axon elongation 201. In addition, cholesterol is responsible for the bilayer curvature required for synaptic vesicle fusion and fission, which is the basis of neurotransmission 202. Cholesterol plays a crucial role in the production and maintenance of many neurotransmitter receptors 203-205 and can specifically interact with these receptors, suggesting another mechanism for regulating neurotransmission 203, 206. Neurogenesis and axonogenesis may be considered as novel hallmarks of cancer associated with cancer progression 207, 208, making them potential targets for tumor therapy. Notably, several studies have demonstrated a strong correlation between cholesterol metabolism and neurogenesis 209-212. Furthermore, Petro et al. found that sequestering cholesterol-induced membrane raft disruption promotes axonogenesis in murine neuroblastoma cells 213.

In summary, cholesterol is abundant in neurons and contributes to the regulation of vital membrane-associated functions of the nervous system. Additionally, cholesterol metabolism is implicated in neurogenesis and axonogenesis, suggesting its potential effects on the innervated niche. However, currently there is no direct evidence to elucidate the correlation between cholesterol metabolism and the innervated niche. Further research is required to address this gap in the field.

## Targeting cholesterol metabolism as a promising antitumor strategy

Ample evidence suggests that cholesterol metabolism is critical for tumor progression. Several preclinical and clinical studies have demonstrated that certain drugs can target cholesterol metabolism and exhibit antitumor effects. Ongoing research continues to explore new drugs that can target cholesterol metabolism in the treatment of cancer (Figure 1 and Table 2).

### Targeting cholesterol biosynthesis

Evidence suggests that dysregulation of the mevalonate pathway drives oncogenic lesions, and targeting the mevalonate pathway to block cholesterol biosynthesis is a feasible antitumor strategy 214, 215. Statins (HMGCR inhibitors) have been extensively studied as repurposed drugs for their antitumor activity. Interestingly, lipophilic statins exhibit higher antitumor activity than hydrophilic statins, indicating their potential superiority in antitumor therapy 216-218. Many epidemiological and clinical studies have shown that statins can reduce the incidence and mortality of tumors 219, 220. We previously summarized that statins can exert antitumor properties in vivo and in vitro through multiple mechanisms 90, and have emerged as promising antitumor agents in various clinical trials 221-223. A randomized controlled trial has shown that fluvastatin suppresses proliferation and increases apoptosis in high grade breast cancer 224. Interestingly, in addition to having direct effects on tumor cells, statins have also been demonstrated to enhance antitumor immunity. Recent studies have shown that simvastatin suppresses lncRNA SNHG29-mediated YAP activation and inhibits PD-L1 expression in CRC 225. Furthermore, statins may maximize efficacy and overcome the shortcomings of conventional cancer therapy 90. However, statin-induced inhibition of the mevalonate pathway drives SREBP activation and the mevalonate pathway-related gene transcription, thereby restoring the mevalonate pathway activity 226, 227. This mechanism may contribute to resistance to statins, potentially accounting for the lack of response to statin therapy in certain patients with cancer 226, 227. Therefore, targeting SREBP is a viable strategy to improve the anticancer effects of statins and overcome drug resistance. Combining statin therapy with SREBP targeting can optimize the antitumor activity. Dipyridamole, which inhibits SREBP cleavage, has been found to enhance the antitumor effects of statins and prevent their resistance by blocking the restorative feedback response of the mevalonate pathway 226, 228-232. Fatostatin and betulin, which are SCAP inhibitors that specifically target SREBP activation, have also been shown to exhibit high antitumor activity 233-238. Furthermore, inhibiting PCSK9 can also inhibit the expression of SREBP. Studies have shown that PCSK9 inhibitors (anti-PCSK9 antibodies and PCSK9 translation inhibitors) combined with simvastatin exert a synergistic antitumor effect in APC/KRAS-mutant CRC 60. Further well-designed clinical trials are required to evaluate the efficacy of combining statins and SREBP inhibitors in cancer therapy.

Other enzymes in the cholesterol biosynthesis pathway may serve as viable targets for pharmacological intervention. Bisphosphonates are inhibitors of the cholesterol biosynthesis pathway that target farnesyl diphosphate synthase (FDPS) to block the production of FPP 239. Preclinical and clinical studies have reported the direct antitumor effects of bisphosphonates 240-243. Furthermore, alendronate, a bisphosphonate used to treat osteoporosis, has been reported to suppress glioblastoma spheres maintenance and activate necrosis-related pathways by inhibiting FDPS 244. SQLE is an oncogene that is frequently overexpressed in various tumors 245. Accumulating evidence indicates that SQLE promotes tumor progression through multiple mechanisms; therefore it is a promising antitumor target 63, 155, 246. Terbinafine, a US Food and Drug Administration (FDA)-approved antifungal drug, has also been investigated for its potential as an antitumor drug by targeting SQLE in multiple studies 62, 155, 247, 248. In addition, Ro 48-8071 can suppress cancer cell proliferation and metastasis by targeting oxidosqualene cyclase (OSC) and preventing the conversion of 2,3-oxidosqualene to lanosterol 249.

### Targeting cholesterol uptake and efflux

LXR is a key transcriptional regulator that maintains cholesterol homeostasis by regulating the function of target genes involved in cholesterol uptake and efflux 250. Several studies have evaluated the potential of targeting cholesterol uptake and efflux as an antitumor strategy, yielding promising outcomes.

Tumor cells upregulate LDLR expression to acquire extrinsic cholesterol and reduce energy consumption during cholesterol biosynthesis 251-253. Bergapten, an LXR agonist, impedes the onset and progression of HCC by regulating the LXR/PI3K/Akt and LXR/IDOL/LDLR pathways and decreasing LDLR expression in a dose-dependent manner 254. Additionally, other LXR agonists, such as LXR-623 and GW3965, induce tumor cell death in GBM via LDLR degradation and ABCA1 cholesterol efflux transporter upregulation 252, 255. Furthermore, GW3965 inhibits melanoma invasion, angiogenesis, and metastasis by inducing apolipoprotein-E (ApoE) expression transcriptionally 256. The LXR agonist T0901317 has shown promise in prostate cancer treatment by inducing apoptosis through ABCG1 expression-mediated cholesterol efflux and subsequent lipid raft disruption 257. In addition to the effects on cancer cells, LXR agonists exert regulatory effects on immune cells. RGX-104, an LXR agonist, has been reported to enhance antitumor immunity by inducing depletion of MDSCs and upregulating ApoE expression, leading to increased activation of CTLs 139. This finding was validated in a phase I clinical trial 139. Interestingly, in addition to LXR activation, LXR repression also affects tumor progression. SR9243, an LXR inverse agonist, induces tumor cell apoptosis by inhibiting the Warburg effect and lipogenesis without causing toxic effects on non-malignant cells 258. Furthermore, in ccRCC, Wu et al. found that both LXR agonist LXR-623 and LXR inverse agonist SR9243 could inhibit tumor cell growth 259. Mechanistically, LXR-623 and SR9243 induce tumor cell apoptosis through distinct mechanisms. LXR-623 reduces intracellular cholesterol levels by upregulating ABCA1 expression and downregulating LDLR expression, whereas SR9243 inhibits intracellular lipogenesis 259. These findings suggest that both LXR activation and repression can influence tumor progression.

PCSK9 regulates cholesterol homeostasis by facilitating LDLR degradation. Recent studies have demonstrated a close association between PCSK9 expression and tumor progression. Clinical evidence suggests that higher PCSK9 expression levels in tumors are negatively correlated with clinical prognosis 260-262, although contradictory findings have been reported 263. PCSK9 has been found to promote tumor progression through multiple mechanisms 59, 72, 73, while also exerting antitumor effects 71, 263. Recently, PCSK9 inhibitors (anti-PCSK9 antibodies and PCSK9 translation inhibitors) have been reported to suppress the growth of APC/KRAS-mutant CRC and exert synergistic antitumor effects with simvastatin 60. Liu et al. reported that PCSK9 inhibition showed promising synergistic antitumor effects in combination with immune checkpoint therapy for tumors 127. Consequently, PCSK9-targeting agents, such as anti-PCSK9 antibodies, vaccines, antisense RNAi, and some drugs (acRoots, lupin peptides, and pseurotin A) have been developed to target the regulation of PCSK9 for potential cancer treatment strategies. 264, 265. Preclinical and clinical investigations are required to evaluate the efficacy and safety of PCSK9 inhibition as a potential tumor therapy. NPC1L1, a cholesterol uptake mediator on the apical surface of enterocytes, facilitates dietary cholesterol absorption 6 and has been linked to colitis-associated tumorigenesis 266. The FDA-approved drug ezetimibe, which inhibits intestinal cholesterol absorption, has demonstrated significant antitumor effects by targeting NPC1L1. Multi-omics analysis revealed that NPC1L1 is an effective therapeutic target for the treatment of PDAC, and ezetimibe inhibits tumor growth without affecting the cytotoxicity of gemcitabine 267. Furthermore, Ezetimibe inhibits tumor angiogenesis in prostate cancer 268, suppresses HCC progression 269, blocks dietary cholesterol absorption in nonalcoholic steatohepatitis (NASH)-driven HCC 270, and enhances antitumor immunity by inhibiting mTORC2 signaling in a CD8^+^ T cell-dependent manner 132. Therefore, targeting intestinal cholesterol absorption is another promising strategy for treating tumors.

### Targeting intracellular cholesterol trafficking

Cholesterol homeostasis depends on intracellular cholesterol trafficking. Studies have shown that inhibition of lysosomal cholesterol release can exert significant antitumor effects. Certain azoles, such as itraconazole, function as intracellular inhibitors of cholesterol trafficking and induce lysosomal cholesterol accumulation by targeting NPC1 and blocking its function 271-274. Thus, itraconazole suppresses tumor growth and angiogenesis by downregulating mTORC1 signaling 273-276. Importantly, the antitumor properties of itraconazole have been further validated in clinical trials 271, 272, 277. Furthermore, astemizole has also been reported to exert antitumor activity by blocking intracellular cholesterol trafficking 278.

In addition to itraconazole and astemizole, other repurposed drugs have demonstrated antineoplastic effects by targeting intracellular cholesterol trafficking, such as selective estrogen receptor (ER) modulators (SERMs), cepharanthine, and leelamine. Tamoxifen, an estrogen receptor modulator, inhibits tumor angiogenesis by blocking intracellular cholesterol trafficking 279. Additionally, cepharanthine, an anti-inflammatory drug, targets NPC1 to inhibit angiogenesis and tumor growth 280. Furthermore, leelamine, a lipophilic diterpene amine, inhibits the tumor cell survival signaling cascade by blocking intracellular cholesterol trafficking 281. More well-designed clinical trials are required to validate the efficacy of these repurposed drugs in cancer treatment.

### Targeting cholesterol esterification

Tumor cells typically exhibit elevated CE levels, suggesting a correlation between cholesterol esterification and tumor progression 282. Evidence suggests that targeting cholesterol esterification is a viable approach for tumor therapy. Avasimibe, a well-studied inhibitor of cholesterol esterification, has been found to effectively suppress tumor cell proliferation by targeting ACAT1 (SOAT1) to inhibit CE production. ACAT1 overexpression in HCC is associated with a poor prognosis 86. Avasimibe has been reported to effectively suppress pancreatic cancer cell proliferation and metastasis by inhibiting cholesterol esterification 88. Bemlih et al. found that avasimibe targets ACAT1 to inhibit tumor cell growth and induce apoptosis in glioblastoma 283, as well as depleting CEs in prostate cancer and impairing the Wnt/β-catenin pathway, which inhibits metastasis 284. Bitter melon extract has been reported to inhibit tumor cell growth in TNBC by downregulating ACAT1 expression 285.

In addition to having direct effects on tumor cells, cholesterol esterification inhibition has also been demonstrated to exert antitumor activity by affecting immune cells. Yang et al. found that avasimibe improves CD8^+^ T cell receptor clustering and immune synapse formation in melanoma by targeting ACAT1, which increases CD8^+^ T cell plasma membrane cholesterol levels, thereby enhancing CD8^+^ T cell-mediated antitumor immunity 117. Avasimibe has also been reported to increase CD8^+^ T cell infiltration in lung cancer 286 and CAR-T cell-mediated antitumor immunity by inhibiting cholesterol esterification 287. Therefore, targeting cholesterol esterification can directly inhibit tumor cells and enhance antitumor immunity, suggesting that targeting cholesterol esterification may be a promising avenue for developing novel therapeutic approaches against cancer.

### Combination therapy

Previously, we provided a summary of the evidence supporting the effectiveness of targeting cholesterol metabolism as monotherapy for tumors. However, in the context of precision medicine, the investigation of drug combination strategies is a crucial area of research. Accumulating evidence suggests that combining a cholesterol metabolism-targeting strategy with other antitumor therapies improves the effectiveness of tumor therapy. This review article explores the potential of such combination strategies targeting cholesterol metabolism for tumor therapy and highlights the advantages of such strategies in alleviating the adverse effects associated with conventional tumor therapy.

Targeting cholesterol metabolism in preclinical studies has demonstrated significant advantages in combination therapies and potential synergies with conventional tumor therapy. Statins, which target cholesterol biosynthesis, have been reported to act synergistically with conventional tumor therapy. For instance, simvastatin has been shown to enhance doxorubicin-induced apoptosis in breast cancer cells 288 and irinotecan-induced growth inhibition and apoptosis in prostate cancer cells 289. Furthermore, atorvastatin increases the radiosensitivity of prostate cancer cells by inhibiting hypoxia-induced HIF-1α protein expression 290. Chen et al. reported that pitavastatin and gemcitabine synergistically inhibited pancreatic cancer cell proliferation by inducing cell cycle arrest, leading to the effective inhibition of tumor growth in nude mouse xenograft models 291. Pitavastatin has been found to increase cytotoxicity and overcome erlotinib resistance in non-small cell lung cancer (NSCLC) when used in combination with erlotinib 292, as well as enhance melanoma cell autophagy and apoptosis when combined with dacarbazine 293. Additionally, Zhang et al. found that lovastatin sensitized gallbladder cancer cells to cisplatin and significantly prolonged the survival of subcutaneous xenograft mice 294. However, statins inhibit the mevalonate pathway, leading to the activation of SREBP and affecting the expression of mevalonate pathway-related genes. This feedback response is a contributing factor to statin resistance 227. Dipyridamole effectively targets SREBP to prevent statin resistance and enhance statin-mediated antitumor activity 226, 228-232. Bisphosphonates (FDPS inhibitors) have also been demonstrated to enhance antitumor effects in combination with statins 295. Jagdev et al. found that the combined use of bisphosphonates and paclitaxel induces apoptosis in breast cancer cells 296. In addition, targeting cholesterol uptake and efflux has shown synergistic effects with conventional antitumor drugs. Chen et al. found that a combination of the LXR agonist GW3965 and EGFR inhibitor afatinib effectively suppressed prostate cancer progression 297. Further research has revealed that GW3965 can increase the sensitivity of NSCLC cells to gefitinib, primarily by inhibiting activation of the Akt-nuclear factor (NF)-κB signaling pathway 298. Additionally, Hu et al. observed that GW3965 could reverse gefitinib resistance in NSCLC by inhibiting vimentin expression 299. Furthermore, the combination of T0901317, an LXR agonist, and gefitinib reduced the migration and invasion of lung cancer cells by inhibiting ERK/MAPK signaling 300. PCSK9 antibodies have been reported to synergistically inhibit tumor growth with programmed cell death protein 1 (PD-1) inhibitors by promoting intratumoral infiltration of cytotoxic T cells, suggesting that PCSK9 inhibition is a feasible strategy for enhancing immune checkpoint therapy 127. In addition, inhibiting drug efflux is also a feasible antitumor strategy. Nelfinavir exhibits synergistic antitumor effects with ezetimibe in multiple myeloma by inhibiting drug efflux transporter ABC subfamily B member 1 (ABCB1) 301. Combining itraconazole, a cholesterol trafficking inhibitor, with 5-FU has shown a synergistic effect in inhibiting the growth of gastric cancer cells 302. Cepharanthine, another cholesterol trafficking inhibitor, has been reported to act synergistically with 5-FU to suppress p53 mutated colorectal cancer cell growth 303. In addition, targeting cholesterol esterification in combination with conventional tumor therapy is a promising strategy. Avasimibe, an ACAT1 inhibitor, exhibits synergistic antitumor effects when used together with gemcitabine in pancreatic cancer 304, enhances the efficacy of chemo-immunotherapy by relieving CD8^+^ T cell suppression 305, and potentially improves anticancer vaccine responses. Avasimibe has been reported to synergize with the KRAS vaccine to enhance antitumor immunity 286. In particular, its combination with a cancer stem cell (CSC)-DC vaccine synergistically inhibited head and neck cancer growth in nude mouse xenograft models 306.

Clinical studies have demonstrated the potential of targeting cholesterol metabolism as adjuvant tumor therapy. In a randomized phase II study, combining simvastatin with gefitinib resulted in increased progression-free survival (PFS) in patients with gefitinib-resistant NSCLC 307. Furthermore, Cantini et al. found that high-intensity statins combined with PD-1 inhibitors improved the prognosis of patients with malignant pleural mesothelioma and advanced NSCLC 308. A randomized controlled trial revealed that pravastatin combined with 5-FU prolonged the survival of patients with advanced HCC, suggesting its potential as an adjuvant therapy for tumors 221. Another randomized controlled trial revealed that atorvastatin is associated with decreased adrenal androgens in the serum and possibly in the prostate, suggesting that atorvastatin may improve the efficacy of androgen deprivation therapy 309. In a randomized, double-blind, placebo-controlled trial, simvastatin improved sensitivity to fluorouracil, adriamycin, and cyclophosphamide therapy in HER2 overexpression locally advanced breast cancer patients 310. Additionally, a cohort study demonstrated that administering statins to lung cancer patients undergoing EGFR-TKI treatment is linked to increased survival rates and has a synergistic effect on antitumor activity 311. Bisphosphonates have shown potential in combination strategies. Epidemiological data indicate that combining bisphosphonates with statins significantly improves survival in patients with cancer 312. A phase 2 clinical trial revealed that combining bisphosphonates with neoadjuvant chemotherapy eliminates disseminated tumor cells more effectively than chemotherapy alone in patients with locally advanced breast cancer 313. Furthermore, the combination of bisphosphonate and chemotherapy significantly reduces the residual invasive tumor size in patients with breast cancer undergoing surgery 314. Additionally, adjuvant bisphosphonates can improve disease-free survival (DFS) in postmenopausal patients with early-stage breast cancer 315. Targeting intracellular cholesterol trafficking in combination with conventional tumor therapy is a promising strategy. Itraconazole combined with conventional tumor therapy has been found to significantly improve the clinical prognosis of cancer patients 316, 317. A retrospective study has shown that the administration of itraconazole in combination with chemotherapy prolongs PFS and overall survival (OS) in patients with refractory ovarian cancer compared to chemotherapy alone 318. In a phase 2 clinical study, itraconazole combined with pemetrexed improved OS in patients with advanced lung cancer compared with pemetrexed alone 319. Therefore, numerous preclinical and clinical studies suggest that cholesterol metabolism-targeting in combination with other standard treatment modalities is a promising antitumor strategy. These evidences may provide a reference for the inclusion of targeting cholesterol metabolism in future tumor therapies.

Importantly, targeting cholesterol metabolism improves the efficacy and mitigates the adverse effects of conventional tumor therapy. Leukopenia is a significant adverse effect of chemotherapy. Atorvastatin was found to be effective in preventing 5-FU chemotherapy-induced leukopenia in an experimental model 320. Cisplatin is widely used in the treatment of various malignant tumors. Despite its therapeutic advantages, this drug has a high level of ototoxicity, causing permanent hearing loss in over 50% of the treated patients 321-325. A combined retrospective and prospective observational study reported that atorvastatin significantly reduced the incidence of cisplatin-induced hearing loss, while maintaining the efficacy of cisplatin 326. Anthracyclines and trastuzumab are frequently used for breast cancer treatment. However, cardiotoxicity associated with these drugs increases the risk of heart failure 327, 328. Based on the findings of multiple clinical studies, statins are considered promising candidates for attenuating anthracycline- and trastuzumab-induced cardiac injury 329-333. These evidences validate the potential of statins in preventing chemotherapy-induced cardiac injury and warrants further clinical trials to investigate their efficacy in preventing cardiotoxicity. Tamoxifen is the primary treatment for ER-positive breast cancers. However, prolonged use of tamoxifen can result in NAFLD 334. When used in combination with tamoxifen, fatostatin, a SREBP inhibitor, has been reported to reduce tamoxifen-induced hepatic lipid accumulation 335. Further research into the molecular mechanisms that target cholesterol metabolism is required to prevent these adverse effects. This could provide a basis for rational combination strategies involving cholesterol metabolism-targeting and antitumor drugs to optimize tumor therapy. In summary, in addition to improving the survival rate of cancer patients, mitigating the adverse effects of conventional tumor therapy is imperative for improving the quality of life of cancer patients.

## Conclusions and perspectives

The role of cholesterol metabolism in tumors has received increasing attention. Therefore, this article provides a summary of the molecular mechanisms by which the dysregulation of cholesterol homeostasis contributes to the hallmarks of cancer. In addition, cholesterol metabolism plays a significant role in the constant communication between tumor cells and their microenvironment. Therefore, this study highlights the diverse effects of cholesterol metabolism on seven specialized microenvironments as well as its involvement in tumor cell-to-TME communication via distinct molecular pathways. Targeting cholesterol metabolism is emerging as a promising antitumor strategy, with drugs capable of exerting antitumor effects by targeting this metabolic pathway. The evidence supporting the use of various antitumor strategies, including targeting cholesterol biosynthesis, uptake and efflux, intracellular trafficking, and esterification, is summarized. Numerous preclinical and clinical studies have suggested that combining conventional tumor therapy with cholesterol-targeting therapies can exert synergistic antitumor effects. Of note, targeting cholesterol enhances the efficacy of conventional tumor therapy and mitigates its adverse effects. These findings suggest that targeting cholesterol metabolism could enhance both the survival and quality of life of patients with cancer.

Despite the initiation of clinical trials for various repurposed drugs that target cholesterol metabolism, certain issues remain unresolved. First, given the low specificity of targeting cholesterol metabolism, which affects both tumor cells and immune cells, improving the specificity of targeting cholesterol metabolism to enhance both direct antitumor effects and antitumor immunity is an urgent problem to be solved. Cholesterol is required for the proliferation and activation of cancer and immune cells 32, 116. In this context, it is particularly important to improve the specificity of targeting cholesterol metabolism. The advancements in nanotechnology enable controlled release of drugs at target sites 336. Targeting cholesterol metabolism combined with nanoparticles may be a potential solution. Second, assessing the suitability of these repurposed cholesterol-targeting drugs in patients with different tumor types. A meta-analysis has shown that statin use reduces mortality in breast cancer patients 218. However, a prospective population-based cohort study suggests that statin use is not associated with prostate cancer recurrence or progression 337. Therefore, further well-designed clinical trials are required to assess the suitability of these repurposed cholesterol-targeting drugs in patients with different tumor types. Third, elucidating the potential adverse effects of these repurposed drugs in cancer patients. For instance, high doses of statins can lead to some adverse effects, with the most common ones being hepatic dysfunction and statin-associated muscle symptoms (SAMS) 338, 339. Nevertheless, statins are generally well tolerated, and adverse effects vary depending on the exact statin used, as well as the dosage and combination with other drugs 340. Therefore, when using repurposed drugs targeting cholesterol metabolism for cancer treatment, adverse effects and other drug interactions should be fully understood, drug dosages should be controlled, and adverse reactions should be monitored. Fourth, evaluating the optimal combination strategy for patients with different tumor types. In a preclinical study, the LXR agonist GW3965 has been reported to increase the sensitivity of NSCLC cells to gefitinib 298. Furthermore, a clinical trial reported that simvastatin improved the efficacy of gefitinib in patients with gefitinib-resistant NSCLC 307. Similarly, several studies have reported that avasimibe and pitavastatin combined with gemcitabine have synergistic antitumor effects in pancreatic cancer 291, 304. A clinical trial reported that pravastatin prolongs survival in patients with advanced HCC treated with 5-FU after catheter arterial embolization 221. However, another clinical trial reported that pravastatin did not improve survival in advanced HCC patients treated with sorafenib 341. Therefore, the optimal combination strategy for patients with different tumor types requires further detailed evaluation. Further well-designed preclinical studies and clinical trials are required to address these unresolved issues.

## Figures and Tables

**Table 1:** Summary of the dysregulation of cholesterol homeostasis and its functions in different cancer types.

Cancer types	Mechanism	Phenotype/effect	References
Breast cancer	Activation of LXRs by inhibiting 27HC catabolism and cholesterol efflux	Tumorigenesis	51
	Copy number amplification of ENSA enhances cholesterol biosynthesis	Tumor progression	32
	Dysregulation of cholesterol homeostasis induces the resistance of metastatic cells to ferroptosis	Enhanced metastatic capacity	7
	Chemokine regulatory loop induces cholesterol biosynthesis	Enhanced metastatic capacity	30
	Enhanced cholesterol biosynthesis pathway	Enhanced metastatic capacity	69
	27HC activates LXR	Enhanced metastatic capacity	77
	27HC promotes an immunosuppressive microenvironment by interacting with immune cells at distal metastatic sites	Enhanced metastatic capacity	78
	SREBP2 regulates osteoclast formation and function	Enhanced metastatic capacity	84
	Enhanced cholesterol biosynthesis pathway	Therapeutic resistance	105
Liver cancer	Enhanced cholesterol biosynthesis activates Hedgehog signaling	Tumorigenesis	39
	Unspliced XBP1 enhances cholesterol biosynthesis by stabilizing SREBP2	Tumorigenesis	41
	Dietary cholesterol induces alterations in gut microbiota and metabolites	Tumorigenesis	64
	LDLR inhibition enhances cholesterol biosynthesis through the MEK/ERK signaling pathway	Enhanced metastatic capacity	70
	SCAP regulates autophagy by influencing AMPK signaling	Therapeutic resistance	110
	Caspase-3-induced SREBP2 activation enhances cholesterol biosynthesis	Therapeutic resistance	111
Prostate cancer	PTEN deletion and PI3K/AKT activation induce cholesterol ester accumulation	Tumor progression	38
	PTEN/p53 deficiency enhances cholesterol biosynthesis by upregulating SQLE expression	Tumor progression	61
	SREBP2 induces transcriptional activation of c-Myc	Enhanced metastatic capacity	83
	27HC enhances the transcriptional activity of androgen receptors and expression of prostate-specific antigens	Therapeutic resistance	92
	HMGCR overexpression	Therapeutic resistance	107
	Cholesterol-rich macrophages transfer cholesterol to tumor cells	Therapeutic resistance	108
Lung cancer	27HC enhances osteoclast differentiation	Enhanced metastatic capacity	81
	Cholesterol induces ABCG2 expression	Therapeutic resistance	96
	HOXB13 induces ABCG1 expression	Therapeutic resistance	97
	Cholesterol promotes ERRα re-expression through the EGFR/Src/Erk/SP1 signaling pathway	Therapeutic resistance	31
	High cholesterol levels in lipid rafts	Therapeutic resistance	109
Gastric cancer	PCSK9 promotes the MAPK signaling pathway by upregulating HSP70	Enhanced metastatic capacity	73
	25HC upregulates TLR2/NF‑κB‑mediated MMP expression	Enhanced metastatic capacity	76
	SOAT1 enhances cholesterol biosynthesis by regulating the expression of SREBP1 and SREBP2	Enhanced metastatic capacity	87
Pancreatic cancer	KIF11 activates mevalonate crosstalk in a SREBP2-dependent manner	Tumor progression	40
	TFCP2 interacts with SREBP2 to synergistically activate cholesterol biosynthesis	Cellular senescence	45
	Cholesterol accumulation in lipid rafts	Therapeutic resistance	91
Colorectal cancer	Overexpression of PSCK9 promotes cholesterol biosynthesis and accumulation of its intermediate GGPP by inhibiting cholesterol uptake	Tumorigenesis	60
	SQLE promotes gut dysbiosis	Tumorigenesis	63
	Cholesterol activates the PI3K/AKT signaling pathway	Tumor progression	37
Melanoma	Ahnak regulates PCSK9 expression	Enhanced metastatic capacity	72
Ovarian cancer	Cholesterol in malignant ascites activates LXRɑ/β	Therapeutic resistance	99
Cholangiocarcinoma	22HC promotes COX-2 expression via a p38 MAPK-dependent mechanism	Tumorigenesis and tumor progression	54
Cervical cancer	Fatty acid synthase regulates cholesterol reprogramming and induces lymphangiogenesis	Enhanced metastatic capacity	89
Bladder cancer	Activation of the mevalonate pathway	Therapeutic resistance	95

**Table 2:** Summary of antitumor drugs that target cholesterol metabolism.

	Target	Drugs	Stage of clinical development	Mechanism	Cancer types	References
**Targeting cholesterol biosynthesis**	HMGCR	Statins	Clinical trial	Atorvastatin inhibits breast cancer proliferation by influencing the expression of cyclin D1 and p27	Breast cancer	222
			Clinical trial	Fluvastatin promotes tumor cell apoptosis	Prostate cancer	223
	SREBP	Fatostatin	Preclinical study	Blocking SREBP-regulated metabolic pathways and androgen receptor signaling networks	Prostate cancer	233
			Preclinical study	Induces tumor cell apoptosis	Endometrial cancer	234
		Betulin	Preclinical study	Induces tumor cell apoptosis	Breast cancer	342
	FDPS	Bisphosphonates	Preclinical study	Suppress tumor angiogenesis through the HIF-1α/VEGF signaling pathway	Breast cancer	343
	SQLE	Terbinafine	Preclinical study	Inhibits cancer cell proliferation and angiogenesis	Oral cancer	248
	OSC	Ro 48-8071	Preclinical study	Suppresses tumor angiogenesis and metastasis	Colorectal cancer, pancreatic cancer	249
**Targeting cholesterol uptake and efflux**	LXR	Bergapten (LXR agonist)	Preclinical study	Inhibits HCC carcinogenesis and progression by regulating the LXR/PI3K/Akt and LXR/IDOL/LDLR pathways and reducing LDLR expression in a dose-dependent manner	Liver cancer	254
		LXR-623 (LXR agonist)	Preclinical study	Promotes LDLR degradation and induces ABCA1 expression	Glioblastoma	252
			Preclinical study	Upregulates ABCA1 expression and downregulates LDLR expression	Kidney cancer	259
		GW3965 (LXR agonist)	Preclinical study	Promotes LDLR degradation and induces ABCA1 expression	Glioblastoma	255
			Preclinical study	GW3965 induces ApoE expression	Melanoma	256
		T0901317 (LXR agonist)	Preclinical study	Upregulates ABCG1 expression	Prostate cancer	257
		RGX-104 (LXR agonist)	Preclinical study and clinical trial	Induces MDSCs depletion and increases CTLs activation by upregulating ApoE expression	Multiple cancer types	139
		SR9243 (LXR inverse agonist)	Preclinical study	Induces tumor cell apoptosis by inhibiting the Warburg effect and lipogenesis	Multiple cancer types	258
	NPC1L1	Ezetimibe	Preclinical study	Inhibits intracellular lipogenesis	Kidney cancer	259
			Preclinical study	Suppresses tumor angiogenesis	Prostate cancer	268
			Preclinical study and clinical trial	Enhances antitumor immunity in a CD8^+^ T cell-dependent manner by inhibiting mTORC2 signaling	Multiple cancer types	132
			Preclinical study	Blocks dietary cholesterol absorption	Liver cancer	270
			Preclinical study	Suppresses tumor angiogenesis	Liver cancer	269
**Targeting intracellular cholesterol trafficking**	NPC1	Itraconazole	Preclinical study	Inhibits angiogenesis and tumor growth	Lung cancer	275
			Preclinical study	Inhibits tumor cell proliferation by inducing autophagy	Glioblastoma	276
			Clinical trial	High-dose has antitumor activity	Prostate cancer	272
			Clinical trial	Antitumor activity	Skin cancer	271
		Astemizole	Preclinical study	Inhibits tumor cell proliferation	Cervical cancer	344
		Tamoxifen	Preclinical study	Inhibits tumor angiogenesis by blocking intracellular cholesterol trafficking	Breast cancer	279
		Cepharanthine	Preclinical study	Inhibits tumor angiogenesis	Multiple cancer types	280
		Leelamine	Preclinical study	Inhibits the signaling cascades that drive tumor cell survival by blocking intracellular cholesterol trafficking	Melanoma	281
**Targeting cholesterol esterification**	ACAT1	Avasimibe	Preclinical study	Inhibits tumor cell proliferation	Liver cancer	86
			Preclinical study	Promotes tumor cell apoptosis by inhibiting cholesterol esterification	Pancreatic cancer	88
			Preclinical study	Induces tumor cell apoptosis	Glioblastoma	283
			Preclinical study	Impairs the Wnt/β-catenin pathway	Prostate cancer	284
			Preclinical study	Enhances antitumor immunity mediated by CD8^+^ T cells	Melanoma	117
			Preclinical study	Enhances antitumor immunity by increasing CD8^+^ T cell tumor infiltration	Lung cancer	286
			Preclinical study	Enhances chimeric antigen receptor-modified T cell-mediated antitumor immunity	Leukemia	287
		Bitter melon extract	Preclinical study	Inhibits tumor cell growth	Breast cancer	285
**Combination therapy**	Chemotherapy + HMGCR	Doxorubicin + simvastatin	Preclinical study	Synergistically promote cancer cell apoptosis	Breast cancer	288
		Anthracycline + statins	Observational clinical cohort study	Associated with a lower incidence of heart failure	Breast cancer	332
		Irinotecan + simvastatin	Preclinical study	Simvastatin enhances irinotecan-induced growth inhibition and apoptosis of cancer cells	Prostate cancer	289
		Gemcitabine + pitavastatin	Preclinical study	Synergistically suppress tumor growth	Pancreatic cancer	291
		Dacarbazine + pitavastatin	Preclinical study	Synergistically promote autophagy and apoptosis in tumor cells	Melanoma	293
		Cisplatin + lovastatin	Preclinical study	Lovastatin sensitizes cancer cells to cisplatin	Gallbladder cancer	294
		Cisplatin + atorvastatin	Clinical trial	Atorvastatin significantly reduces the incidence of cisplatin-induced hearing loss without reducing the efficacy of cisplatin	Head and neck cancer	326
		5-FU + pravastatin	Clinical trial	Prolongs the survival of patients with advanced HCC	Liver cancer	221
		5-FU + atorvastatin	Preclinical study	Atorvastatin effectively prevents leukopenia secondary to experimental 5-FU chemotherapy	Not reported	320
	Radiotherapy + HMGCR	Radiotherapy + atorvastatin	Preclinical study	Atorvastatin enhances the radiosensitivity of prostate cancer cells by inhibiting hypoxia-induced HIF-1α protein expression	Prostate cancer	290
	SREBP + HMGCR	Dipyridamole + statins (Atorvastatin, simvastatin, and rosuvastatin)	Preclinical study	Dipyridamole enhances the antitumor activity of statins and prevents their resistance	Breast cancer	226
		Dipyridamole + fluvastatin	Preclinical study	Dipyridamole enhances apoptosis in statin-insensitive tumor cells	Prostate cancer	229
	Targeted therapy + HMGCR	Erlotinib + pitavastatin	Preclinical study	Synergistcally enhances cytotoxicity and overcome erlotinib resistance	Lung cancer	292
		Gefitinib + simvastatin	Clinical trial	Increases PFS in patients	Lung cancer	307
		Trastuzumab+ statins (Atorvastatin, simvastatin, rosuvastatin, and pravastatin)	Randomized Controlled Trial	Statins effectively reduce trastuzumab- induced cardiotoxicity	Breast cancer	331
	Immunotherapy + HMGCR	PD-1 inhibitors+ statins (Atorvastatin, simvastatin, rosuvastatin, and others)	Clinical trial	High-intensity statins can enhance the clinical activity of PD-1 inhibitors	Lung cancer	308
	FDPS + HMGCR	Zoledronic acid + statins (Atorvastatin, simvastatin, and rosuvastatin)	Preclinical study	Synergistically exert antitumor effects	Breast cancer	295
	Chemotherapy + FDPS	Paclitaxel + Zoledronic acid	Preclinical study	Synergistically promote tumor cell apoptosis	Breast cancer	296
		Epirubicin + docetaxel + bisphosphonates	Clinical trial	Significantly enhances the clearance of disseminated tumor cells in patients with locally advanced breast cancer compared with chemotherapy alone	Breast cancer	313
	Targeted therapy + LXR	Afatinib + GW3965	Preclinical study	Synergistically inhibit tumor progression	Prostate cancer	297
		Gefitinib + GW3965	Preclinical study	GW3965 enhances the sensitivity of NSCLC cells to gefitinib by inhibiting activation of the Akt‑NF‑κB signaling pathway	Lung cancer	298
		Gefitinib + GW3965	Preclinical study	GW3965 reverses gefitinib resistance in NSCLC by inhibiting vimentin expression	Lung cancer	299
		Gefitinib + T0901317	Preclinical study	Suppresses the migration and invasion of tumor cells by inhibiting the ERK/MAPK signaling	Lung cancer	300
	Immunotherapy + PCSK9	PD-1 inhibitors + PCSK9 antibodies	Preclinical study	PCSK9 antibodies synergistically inhibit tumor growth with PD-1 inhibitors by promoting the intratumoral infiltration of cytotoxic T cells	Multiple cancer types	127
	Chemotherapy + NPC1	5-FU + itraconazole	Preclinical study	Synergistically inhibit tumor cell growth	Gastric cancer	302
		Pemetrexed + itraconazole	Clinical trial	Improved OS in patients with advanced lung cancer compared with pemetrexed alone	Lung cancer	319
		5-FU + cepharanthine	Preclinical study	Synergistically inhibit the growth of colorectal cancer cells containing p53 mutations	Colorectal cancer	303
	Chemotherapy + ACAT1	Gemcitabine + avasimibe	Preclinical study	Synergistically exert antitumor effects	Pancreatic cancer	304
	Chemoimmunotherapy + ACAT1	Paclitaxel + immunoadjuvant αGC + avasimibe	Preclinical study	Avasimibe enhances the antitumor activity of chemo-immunotherapy by relieving CD8^+^ T cell suppression	Melanoma	305
	Anticancer vaccines + ACAT1	CSCs-DC vaccine + avasimibe	Preclinical study	Synergistically inhibit tumor cell growth	Head and neck cancer	306

## Abbreviations

ANO1: Anoctamin 1
TME: Tumor microenvironment
HMG-CoA: 3-hydroxy-3-methyl-glutaryl-CoA
IPP: Isopentenyl pyrophosphate
GPP: Geranyl pyrophosphate
FPP: Farnesyl pyrophosphate
SREBP2: Sterol regulatory element-binding protein 2
LXR: Liver X receptor
ER: Endoplasmic reticulum
SCAP: SREBP cleavage-activating protein
INSIG: Insulin-induced gene
S1P: Site-1 protease
S2P: Site-2 protease
HMGCR: HMG‑CoA reductase
LDL: Low-density lipoprotein
LDLR: Low-density lipoprotein receptor
NPC1L1: Niemann-Pick type C1-like 1
NPC: Niemann-Pick C
PCSK9: Proprotein convertase subtilisin/kexin type 9
SRE: Sterol regulatory elements
CEs: Cholesterol esters
ACAT: Acyl-CoA: cholesteryl acyltransferase
LDs: Lipid droplets
ABC: ATP-binding cassette
ABCA: ATP-binding cassette subfamily A member
ABCB: ATP-binding cassette subfamily B member
ABCG: ABC subfamily G member
HDL: High-density lipoprotein
CRC: Colorectal cancer
ENSA: Gene alpha-endosulfine
TNBC: Triple-negative breast cancer
PDAC: Pancreatic ductal adenocarcinoma
XBP1: X-box binding protein 1
HCC: Hepatocellular carcinoma
TFCP2: Transcription factor CP2
27HC: 27-hydroxycholesterol
RCC: Renal cell carcinoma
ccRCC: Clear cell renal cell carcinoma
ZMYND8: Zinc finger MYND-type containing 8
ER-BC: Estrogen receptor-negative breast cancer
GPER: G protein-coupled estrogen receptor
GGPP: Geranylgeranyl diphosphate
SQLE: Squalene epoxidase
CRPC: Castration-resistant prostate cancer
GC: Gastric cancer
CH25H: Cholesterol 25-hydroxylase
MMP: Matrix metalloproteinase
SOAT1: Sterol O-acyltransferase 1
CRABP-II: Cellular retinoic acid-binding protein II
AR: Androgen receptor
5-FU: 5‑fluorouracil
PD-L1: Programmed death ligand 1
CRAC: Cholesterol-recognition amino acid consensus
ER+: Estrogen receptor-positive
EGFR-TKIs: Epidermal growth factor receptor tyrosine kinase inhibitors
NSCLC: Non-small cell lung cancer
TIICs: Tumor-infiltrating immune cells
TIME: Tumor immune microenvironment
NK: Natural killer
TAMs: Tumor-associated macrophages
MDSCs: Myeloid-derived suppressor cells
DCs: Dendritic cells
TILs: Tumor-infiltrating lymphocytes
CTLs: Cytotoxic T lymphocytes
MHC-I: Major histocompatibility complex class I
CAR: Chimeric antigen receptor
TCR: T-cell receptor
IL-9: Interleukin-9
NKT: Natural killer T
mTORC2: Mammalian target of rapamycin complex 2
CXCR2: CXC chemokine receptor 2
HIF-1α: Hypoxia-inducible factor-1α
GBM: Glioblastoma multiforme
GPR: G-protein-coupled receptor
CCR7: CC chemokine receptor-7
ROS: Reactive oxygen species
DNMT3A: DNA methyltransferase 3A
RECK: Reversion-inducing-cysteine-rich protein with Kazal motifs
HIFs: Hypoxia-inducible factors
PKB: Protein kinase B
CAFs: Cancer-associated fibroblasts
YAP: Yes-associated protein
ECM: Extracellular matrix
EMT: Epithelial-mesenchymal transition
CXCL1/2/8: C-X-C motif chemokines 1/2/8
CCL2/7: C-C motif chemokines 2/7
TAZ: PDZ-binding motif
TFEB: Transcription factor EB
APMAP: Adipocyte plasma membrane-associated protein
ERK1/2: Extracellular-regulated protein kinase 1/2
NAFLD: Non-alcoholic fatty liver disease
FDPS: Farnesyl diphosphate synthase
FDA: Food and Drug Administration
OSC: Oxidosqualene cyclase
ApoE: Apolipoprotein-E
NASH: Nonalcoholic steatohepatitis
SERMs: Selective estrogen receptor modulators
NF: Nuclear factor
PD-1: Programmed cell death protein 1
CSC: Cancer stem cell
PFS: Progression-free survival
DFS: Disease-free survival
OS: Overall survival
SAMS: Statin-associated muscle symptoms
